# Traditional Sheep Consumption by Navajo People in Cameron, Arizona

**DOI:** 10.3390/ijerph16214195

**Published:** 2019-10-30

**Authors:** Tommy Rock, Ricky Camplain, Nicolette I. Teufel-Shone, Jani C. Ingram

**Affiliations:** 1 School of Earth & Sustainability, Northern Arizona University, Flagstaff, AZ 86011, USA; rockt92@gmail.com; 2 Center for Health Equity Research, Northern Arizona University, Flagstaff, AZ 86011, USA; Ricky.Camplain@nau.edu (R.C.); Nicky.Teufel@nau.edu (N.I.T.-S.); 3 Department of Chemistry & Biochemistry, Northern Arizona University, Flagstaff, AZ 86011, USA

**Keywords:** Navajo, traditional food, consumption, abandoned mines

## Abstract

Over 500 abandoned uranium mines are located on the Navajo Reservation. Different pathways of environmental uranium exposure have been studied with respect to the Navajo people including water, soil, and plants; however, uranium exposure from traditional Navajo food, specifically mutton (sheep), has not been reported. This study focuses on mutton consumption in the small community of Cameron, Arizona, located in the southwestern region of the Navajo Nation and initiated after community members expressed concern with the uranium exposure of their sheep. Preliminary investigation into the presence of uranium in sheep raised near Cameron showed elevated uranium levels in the kidneys the sheep tested. The goal of this study is to investigate mutton consumption among the Navajo living in Cameron. Mutton is a traditional food of the Navajo, but consumption practices are not well documented. An important aspect of determining the extent of exposure through food consumption is to assess the frequency of consumption. The results of this study indicate the Cameron participants consume mutton most commonly at family gatherings or celebrations. The survey suggests that less mutton is consumed now compared to the past, and there is concern that contaminated mutton may change traditional ceremonies.

## 1. Introduction

**Figure 1:**
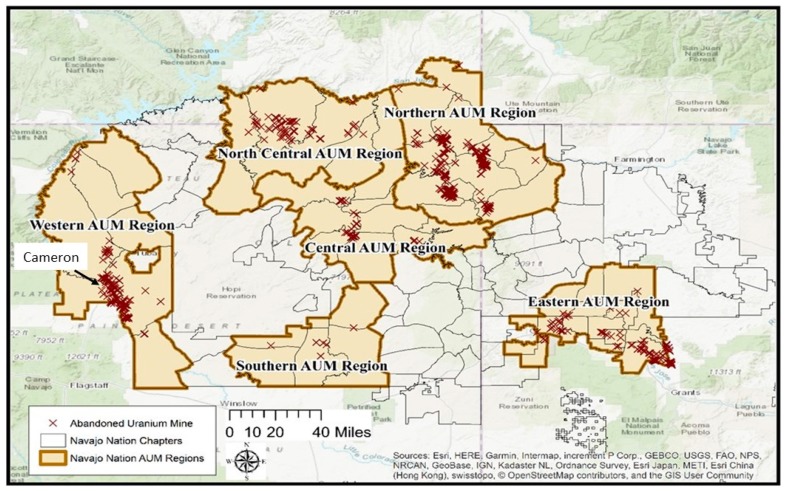
Map of the Navajo Nation illustrating the abandoned uranium mine locations.

The Navajo Nation is the largest contiguous Native American reservation in the continental United States. Located within the Four Corners region of the American Southwest, its borders span 71,000 square kilometers across Arizona, New Mexico, and Utah. The Navajo Nation is recognized by the United States’ government as a sovereign nation, though the United States retains plenary power, and is separated into 110 tribal Chapters governed through five management Agencies. The Cameron chapter is located on the Navajo Reservation in northern Arizona, approximately 50 miles north of Flagstaff, AZ and 35 miles east of the Grand Canyon (Figure 1). According to the 2010 United States census, the Cameron Chapter had a population of 885 with a land area of 238,523 acres [1]. Of the 885 Cameron residents, 832 of these are American Indian/Alaska Native. The gender breakdown of the Cameron population is 51% female/49% male. The adult population (people over the age of 18 years) is 624 community members. The area is in the Painted Desert and on the East Kaibab Monocline [2]. Uranium was discovered in the Chinle Formation of the Upper Triassic Period [3], specifically in the Petrified Forest Member [4]. Past uranium mining in the Cameron Chapter extends back to 1952, leaving a legacy of 98 open pit abandoned uranium mines located within the Cameron Chapter boundaries documented by the United State Geological Survey [3]. Figure 1 shows a map of the Navajo Nation; the Cameron Chapter is located in the western region of the Navajo Nation. The abandoned mines are indicated by X’s on the map.

Uranium is both radioactive and is a heavy metal. Chemically, uranium targets certain organs in the body such as the kidney and liver, causing damage to these target organs [5,6]. It has been shown that uranium and its radioactive decay products can be absorbed through contaminated food sources [7], particularly by accumulation in the bone, which potentially can cause bone cancer. Several studies have shown chronic uranium exposure through consumption of contaminated water can act as a nephrotoxin [8,9] and can affect the stomach [10] as well as accumulate in the bone [11]. Another study suggested that uranium could also target the brain and teeth [12]. Chronic exposure to uranium can also affect the central nervous system [13].

The pathway of exposure to environmental uranium for humans living on the Navajo Reservation through vegetation [14], water [15], and soil [16] have been previously studied [17]. However, livestock and wildlife have not yet been studied to determine the impact of exposure to uranium on the Navajo Reservation. Off the reservation, the Oak Ridge National Laboratory (ORNL), located in eastern Tennessee, discovered wildlife (insect, birds, and mammals) living near ORNL had elevated levels of radionuclides in tissue samples compared to other similar wildlife living in other areas of Tennessee [18]. These findings were supported by other studies [19,20], which found that wildlife in closer proximity of contaminated radioactive sites had higher radioactivity levels in their tissues compared to animals living in areas further away.

Uranium was found in sheep blood at higher concentrations in areas where depleted uranium ammunition was used by the allied forces during the 1999 NATO attacks in Southern Serbia [21]. Previous research illustrated a pathway of exposure to humans from contaminated animals in areas of mining in South Africa, which could pose a possible public health risk [20]. Navajo people traditionally eat sheep as a part of their diet [22]; sheep are also important in traditional ceremonies [23]. Consumption of sheep exposed to uranium, such as those who may graze near one of the 98 open pit uranium mines near Cameron, AZ, may be a public health risk.

One study from Bosshard [24] described exposure to uranium through food. The study indicated that the range of uranium exposure was between 1–3 µg/person/day in regions of no uranium mining and as high as 9 µg/person/day in regions of uranium mining. A separate study from Canada investigated moose grazing near uranium mines and concluded that the Indigenous People should not consume the kidneys as the levels of uranium were found to be 2–3 times higher than moose grazing in the control area [25]. Uranium exposure and maximum contaminant limits have been set for uranium consumption in drinking water at 30 µg/L [26]; however, regulations on maximum uranium exposure levels in food have not been determined.

Studies have shown a gender difference in uranium accumulation. Males tend to accumulate more uranium in the kidney [6] and gastrointestinal tract [27] compared to females. The most vulnerable population to uranium accumulation is infants, which may exceed adults by three-fold [28].

The Navajo people have felt the ramifications of uranium mining. Today there are 523 abandoned uranium mines located on the Navajo Reservation with only one having been remediated [29]. Another concern from uranium contamination is the groundwater contamination from historical uranium mining [15]. The Navajo Nation Tribal Council passed a resolution in 2007 that prohibited further uranium mining on the Navajo Nation, which honors the traditional values of the Navajo Nation to protect Mother Earth [30]. The environmental uranium exposures of the Navajo people are unique because of previous mining activity and the lifestyle of the Navajo people.

Multiple Navajo communities requested information from the Northern Arizona University (NAU) research team concerning uranium levels in their traditional food source, specifically sheep. Since the 1600s, sheep have been important to the Navajo in a variety of ways. Mutton fed Navajo families, wool kept them warm, and the sale of blankets woven by women of the family group provided cash from the local trader [31]. This study was requested by the Cameron community and presents an opportunity for the NAU research team to assist the Navajo people to obtain scientific information that is critical for their health and sovereignty. By responding to this request, the NAU research team can continue building a trusting relationship between the Navajo Nation and researchers, while gaining insights into environmental uranium exposure. In a sheep study of bioaccumulation of uranium in sheep in Cameron, Arizona, the results suggest elevated levels of uranium in the kidneys of the sheep raised in the southwestern region of the Navajo Reservation ([32], see Supplemental Material). In this study, kidneys from five sheep raised in Cameron were compared to kidneys from five sheep raised in Eager, Arizona, the control site, located 80 miles south of the Navajo Reservation and 100 miles south of the closest abandoned uranium mine. The study indicated that the average uranium level in the Cameron sheep kidney was 120 ppb while the average uranium level in the Eager sheep kidney was 30 ppb; this is a four-fold increase in uranium content. The concern of the Cameron community to eating mutton from meat with potentially elevated levels of uranium is the foundation for the research reported here. An important question in addressing this concern is with regard to the mutton consumption practices among the Navajo. Thus, the goal of the study is to investigate the consumption of mutton in the community of Cameron by gender and age groups. Additionally, questions were asked to assess the perceptions of participants regarding the importance of mutton in traditional ceremonies. Gaining an understanding of the amount of mutton raised on the Navajo Reservation and consumed by the Navajo people is key to providing guidance for safe consumption of this important traditional food.

## 2. Materials and Methods

The research was conducted in Cameron, AZ, located in northern Arizona in the southwestern area of the Navajo Nation. The mutton consumption survey was administered to a convenience sample of 72 members of the Cameron Chapter. Prior to the study, the Chapter approved the research by voting on a resolution of support for the survey. Additionally, approval was granted by the Navajo Nation Western Agency and the Tuba City Health Board. The study protocol, survey, and consent form were approved by the NAU Institutional Review Board and the Navajo Nation Human Research Review Board (see Supplementary Materials for approval documentation).

The Diné Hataalii Association (Navajo Medicine Men) was consulted to provide insights into Navajo culture to shape policies around sheep consumption. This approach was taken to assure cultural sensitivity around issues shaping the lives of the Navajo people. The study focused on adapting the Indigenous Health Indicator [33] developed by the Swinomish Tribe. This entailed meeting with the Diné Hataalii Association members to get their input on the relationship between Navajo consumption of potentially contaminated sheep and its relationship to the Navajo Fundamental Laws [34]. To determine the appropriateness of the questions, the survey was beta tested among 15 Cameron community members at the 14 December 2016, Cameron Monthly Chapter meeting. To assess readability, cultural appropriateness, and Face Validity, community members provided detailed input on question readability, perceived cultural appropriateness of individual questions, and what they believed the questions measured.

Surveys were conducted at social gatherings in Cameron, Arizona, between June and October 2017. The first event where the survey was conducted was a Veterans’ celebration. Afterward, the Cameron Chapter had a social gathering of commodity food distribution along with a Health Fair event for community members to attend. A second event was the Cameron Days Celebration that occurs during the Navajo Nation Western Agency Fair.

The community was introduced to a questionnaire about personal mutton consumption and perspective of the role of mutton in traditional Navajo Culture. Descriptive characteristics, including gender, age, community, tribal affiliation, number of adults and children in their household, and if they owned sheep, were self-reported. Participants were asked about their lifetime pattern of mutton consumption as well as their views on the impact to traditional ceremonies. Participants were also asked at which events they ate mutton such as family, community, or ceremonial gatherings and if they viewed the mutton they consume as a health risk due to uranium.

Descriptive characteristics in the questionnaire were presented as counts and percentages. Mutton ceremony perceptions and eating habits among survey respondents were presented as counts and percentages by gender and age groups. Counts and percentages of the frequency of consumption and preparation of mutton were presented overall. Responses were not mutually exclusive and percentages may not always add up to 100%. All analyses were conducted using SAS V9.4 (SAS Inc., Cary, NC, USA).

## 3. Results

Table 1 presents the demographic information of the participants who completed the sheep consumption survey. Of the 72 participants who completed the survey, most were 40 years and older (72%), female (60%), identified as Navajo (96%), and were from the Cameron Chapter (57%). The number of participants not living in the Cameron Chapter (43%) is identified as people who grew up in Cameron as children but have moved away and lived elsewhere as an adult. These participants go back to Cameron for community events to show their support for their childhood community. According to the 2010 Census, there were 624 adults living in Cameron [1]; thus, approximately 6.6% of the Cameron adult population participated in the survey. There was an average of 2.6 adults and 1.5 children in the households with only 25% of participants self-reported owning sheep. This table indicates that the least represented age group in this study was the 18–25-year-old group. As to the gender of the participants, there were more women than men. Most of the participants identify with the community of Cameron but resides elsewhere.

Table 2 describes the importance of mutton in traditional cultural environment. While 93% of participants believe mutton is important for Navajo traditions, 58% of all participants and 49% of participants that reported mutton is important for Navajo traditions also believe ceremonies would not change if the mutton were not available. Many of the female participants (54%) do think that ceremonies will change, while 25% of the male participants do not think the ceremonies will change. The results suggest a gender difference in perception of change in ceremonies if the mutton is contaminated. There were also differences in responses by age group. The majority (86%) of younger participants (18–25 years) believed that ceremonies would be changed if there was no mutton, compared to older age groups (>40 years). Additionally, slightly more than half (52%) of the participants believe it would be impossible to have a ceremony if the mutton was contaminated. Again, a difference between the gender responses was observed with a higher percentage of female participants responding positively to ceremonies being impossible to perform without mutton compared to the male participants. In contrast to whether ceremonies would be changed, most (75%) of older adults (>70 years) believe that some ceremonies would be impossible to perform if there was no mutton compared to younger participants’ (<55 years) response that ceremonies would still be possible (57%) with no mutton.

Table 3 illustrates perception of safety with respect to eating mutton from sheep that have grazed around abandoned uranium mines near Cameron, AZ. Almost half (48%) of participants did not think or worry whether it was safe to eat mutton while 23% percent did. Participants were also asked at what events do they eat sheep. About 80% of participants consumed mutton during family gatherings while only 25% consumed mutton during regular meals. Additionally, 54% self-reported consuming mutton at ceremonies and 53% consumed mutton at holiday events. In terms of lifetime mutton consumption habits, 73% of the surveyed population agree that people ate more mutton when they were younger compared to today. Only 19% do not think people ate more mutton in the past.

Table 4 describes the preparation-serving styles of different organs and tissues of the sheep. The results in Table 4 show that the majority of survey participants consume many different parts of a sheep. The cooking preparation of mutton is the most likely to be stewed or grilled for all the different sheep tissues.

Table 5 describes the frequency of consumption of the different organs and tissues of the sheep. The majority of participants who reported eating the specific organ or tissues reported consuming blood sausage, mutton stew, roasted mutton sandwiches, ribs, hind legs, Achii once a month (26–32%) or every few months (50–60%). Few participants reported eating any part of the sheep every day (0–6% or 1–2 individuals).

## 4. Discussion

In the Cameron Chapter, consumption of mutton occurs most often during family gathering events. This practice may explain why the majority of participants believe mutton is important for traditions. There were more female participants than male in this study, similar to the participation in a study from the University of New Mexico called the Diné Network for Environmental Health (DiNEH) Project [35]. The DiNEH study was focused on kidney disease associated with uranium exposure on Navajo Nation. In the study reported here, there were fewer participants from the 18–25 years age group, which was also similar to participant response in the DiNEH Project. The results of the study reported here suggest that if the mutton is contaminated, all members of the family will have some levels of exposure to uranium through eating mutton with the older population (>40 years) eating mutton on a more frequent basis. However, the health risk associated with consuming uranium-contaminated mutton is unknown at the present time. The purpose of this study was to initiate interactions with the community to learn more about their mutton consumption practices as well as gain an understanding of the connection between mutton and cultural practices. Additional work is needed to determine uranium exposure to the Navajo people through mutton consumption. The next step in this research is to test different organs and tissues of the sheep prior to and after cooking in specific ways to estimate the exposure to uranium. A more detailed study of the actual amounts of mutton consumed as well as the specific frequencies of consumption is needed to determine exposure due to mutton consumption. Based on discussions with the participants in the study, a rough estimate is that between one half to one pound of meat is consumed at any given meal. Most families participate in at least one gathering (such as a birthday) per month where mutton is served. More work is needed to better define the exposure to uranium from mutton consumption.

In terms of traditional Navajo ceremonies, the survey suggests a difference of opinion between genders. A majority of female participants think that ceremonies will change, while most male participants do not think it will. In Navajo ceremonies, mutton is the main dish. Mutton’s role in ceremonies in the future maybe modified if contamination is truly an issue. Navajo Elders and Traditional Knowledge Holders need to voice their opinion on the impact on culture and ceremonies. The potential of uranium exposure through food consumption is likely to be associated with areas of past uranium mining activities on the Navajo Nation. Clearly, additional investigation of other areas on the Navajo reservation should be pursued. Culturally relevant traditional food consumption should be addressed through the combination of results from studies as is reported here and consultation with Navajo Elders [36]. The approach taken in this study is to collaborate with Navajo Elders in order to shape how developing policies can safely address public health while preserving the Navajo cultural use of mutton.

## 5. Conclusions

The results of the mutton survey administered in this study suggest Navajo ceremonies may be altered or threatened due to contamination of mutton, a traditional staple food of the Navajo people. The request by the community of Cameron to investigate sheep contamination illustrates the concern for mutton consumption in uranium mining communities on the Navajo Nation. The frequency of mutton consumption is most often around family functions, although holidays also represent an important time of consumption. The results from this study suggest that only a few Navajo people consume mutton daily and that current mutton consumption is lower compared to the past. This study was conducted in one community on the Navajo Nation; thus, additional Navajo communities should be surveyed to understand if mutton consumption patterns are similar or different throughout the Navajo Nation. The paucity of studies on uranium exposure through food consumption compared to studies on uranium exposure through drinking water points to the need for additional research. A study in Canada echoed the sentiment that research is needed with respect to Indigenous food consumption in relation to uranium exposure [25]. Collaboration between researchers and Navajo Elders is a useful approach to creating policies that can address cultural sensitivity around traditional food use of mutton.

## Figures and Tables

**Table 1:** Demographic information for participants who completed the sheep survey, 2017 (*n* = 72).

Characteristic	*N* (%) or Mean ± SD
Age group	
18–25 years	7 (9.7)
26–39 years	13 (18.1)
40–55 years	17 (23.6)
56–70 years	21 (29.2)
>70 years	14 (19.4)
% Female	43 (59.7)
Community	
Cameron	41 (56.9)
Tuba City	12 (16.7)
Other	19 (26.4)
% Navajo	69 (95.8)
Adults in household	2.6 ± 1.5
Children in household	1.5 ± 1.7
Sheep (% yes)	18 (25)

Abbreviations: Standard deviation (SD).

**Table 2:** Mutton ceremony perceptions among participants who completed the sheep survey, 2017 (*n* = 72).

Question	Response, *N* (%)							
	All Participants	Gender		Age Group (years)				
		Men	Women	18–25	26–39	40–55	56–70	>70
**Do you think mutton is important for traditions?**								
Yes	66 (93.0)	25 (89.3)	41 (95.4)	7 (100.0)	12 (92.3)	16 (94.1)	20 (95.2)	11 (84.6)
No	5 (7.0)	3 (10.7)	2 (4.6)	0 (0.0)	1 (7.7)	1 (5.9)	1 (4.8)	2 (15.4)
Missing *	1	1	0	0	0	0	0	1
**Would ceremonies be changed if there was no mutton?**								
Yes	30 (42.3)	7 (25.0)	23 (53.5)	6 (85.7)	6 (46.2)	6 (35.3)	8 (38.1)	4 (30.8)
No	41 (57.7)	21 (75.0)	20 (46.5)	1 (14.3)	7 (53.8)	11 (64.7)	13 (61.9)	9 (69.2)
Missing *	1	1	0	0	0	0	0	1
**Would some ceremonies be impossible to perform if there was no mutton?**								
Yes	36 (52.2)	9 (34.6)	27 (62.8)	4 (57.1)	3 (23.1)	9 (52.9)	11 (55.0)	9 (75.0)
No	33 (47.8)	17 (65.4)	16 (37.2)	3 (42.9)	10 (76.9)	8 (47.1)	9 (45.0)	3 (25.0)
Missing *	3	3	0	0	0	0	1	2

* Missing not included in percent.

**Table 3:** Mutton eating habits among participants who completed the sheep survey, 2017 (*n* = 72).

Question	Response, *N* (%)							
	All Participants	Gender		Age Group (years)				
		Men	Women	18–25	26–39	40–55	56–70	>70
**Do you think or worry whether it is safe to eat mutton?**								
Yes	23 (32.4)	7 (25.0)	16 (37.2)	2 (28.6)	2 (15.4)	3 (17.7)	7 (33.3)	9 (69.2)
No	34 (47.9)	18 (64.3)	16 (37.2)	4 (57.1)	9 (69.2)	10 (58.8)	9 (42.9)	2 (15.4)
Sometimes	14 (19.7)	3 (10.7)	11 (25.6)	1 (14.3)	2 (15.4)	4 (23.5)	5 (23.8)	2 (15.4)
Missing *	1	1	0	0	0	0	0	1
At what events to you eat sheep? **								
Regular meal	18 (25.0)	5 (17.2)	13 (30.2)	0 (0.0)	2 (15.4)	6 (35.3)	4 (19.1)	6 (42.9)
Ceremony	39 (54.2)	15 (51.7)	24 (55.8)	6 (85.7)	7 (53.9)	9 (52.9)	8 (38.1)	9 (64.3)
Holiday	38 (52.8)	9 (31.0)	29 (67.4)	4 (57.1)	9 (69.2)	7 (41.2)	12 (57.1)	6 (42.9)
Family gathering	60 (83.3)	22 (75.9)	38 (88.4)	7 (100.0)	11 (84.6)	14 (82.3)	18 (85.7)	10 (71.4)
Community event	30 (41.7)	10 (34.5)	20 (46.5)	1 (14.3)	6 (46.2)	6 (35.3)	12 (57.1)	5 (35.7)
**Do you think people eat more mutton in your childhood compared to today?**								
Yes	52 (73.2)	20 (71.4)	32 (74.4)	6 (85.7)	7 (53.9)	10 (58.8)	17 (81.0)	12 (92.3)
No	19 (26.8)	8 (28.6)	11 (25.6)	1 (14.3)	6 (46.1)	7 (41.2)	4 (19.0)	1 (7.7)
Missing *	1	1	0	0	0	0	0	1

* Missing not included in percent; ** Participants may choose more than one response.

**Table 4:** Preparation of mutton among participants who completed the sheep survey, 2017 (*n* = 72).

Part of Sheep Prepared	Number of Participants	How Sheep was Prepared					
		Roasted	Grilled	Fried	Stewed	Boiled	Other
	*N* (%)	*N* (%) *					
Blood sausage	52 (72.2)	3 (5.8)	2 (3.9)	1 (1.9)	1 (1.9)	46 (88.5)	0
Mutton stew	63 (87.5)	2 (3.2)	7 (11.1)	2 (3.2)	20 (31.8)	43 (68.3)	1 (1.6)
Roasted mutton Sandwich	59 (81.9)	20 (33.9)	37 (62.7)	4 (6.8)	0	2 (3.4)	0
Mutton ribs	60 (83.3)	20 (33.3)	40 (66.7)	3 (5.0)	0	2 (3.3)	0
Roasted mutton meat	60 (83.3)	26 (43.3)	31 (51.7)	5 (8.33)	5 (8.3)	2 (3.3)	0
Hind leg	57 (79.2)	15 (26.3)	22 (38.6)	5 (8.8)	16 (28.1)	22 (38.6)	0
Intestines (Achii)	53 (73.6)	14 (26.4)	33 (62.3)	12 (22.6)	0	3 (5.7)	0
Liver	53 (73.6)	15 (28.3)	25 (47.2)	17 (32.1)	0	2 (3.8)	0
Heart	45 (62.5)	8 (17.8)	16 (35.6)	17 (37.8)	0	8 (17.8)	0
Kidneys	47 (65.3)	12 (25.5)	23 (48.9)	15 (31.9)	0	4 (8.5)	0
Lungs	38 (52.8)	7 (18.4)	12 (31.6)	14 (36.8)	0	8 (21.1)	0
Esophagus	18 (25.0)	5 (27.8)	5 (27.8)	6 (33.3)	0	4 (22.2)	0
Hoof	15 (20.8)	6 (40.0)	8 (53.3)	1 (6.7)	0	3 (20.0)	1 (6.7)
Skin	12 (16.7)	3 (25.0)	8 (66.7)	1 (8.3)	0	2 (16.7)	0
Head	46 (63.9)	33 (71.7)	10 (21.7)	2 (4.4)	0	3 (6.5)	0
Tongue	39 (54.17)	25 (64.1)	8 (20.5)	2 (5.1)	0	5 (12.8)	0
Eyes	39 (54.2)	29 (74.4)	7 (18.0)	1 (2.6)	0	3 (7.7)	0
Ears	24 (33.3)	15 (62.5)	7 (29.2)	1 (4.2)	0	1 (4.2)	0
Stomach	45 (62.5)	9 (20.0)	9 (20.0)	9 (20.0)	2 (4.4)	24 (53.3)	0

* Percent is of the number of participants who consumed the respective part of the sheep; not mutually exclusive (percentages will not always add up to 100%).

**Table 5:** Frequency of consumption among participants who completed the sheep survey, 2017 (*n* = 72).

Part of Sheep Consumed	Number of Participants	Frequency of Consumption					
		Every Day	Every Week	Every Month	Every Few Months	Once a Year	Missing
	*N* (%)	*N* (%) *					
Blood sausage	52 (72.2)	1 (1.9)	3 (5.8)	14 (26.9)	26 (50.0)	6 (11.5)	2 (3.8)
Mutton stew	63 (87.5)	2 (3.2)	8 (12.7)	20 (31.8)	32 (50.8)	0	1 (1.6)
Roasted mutton sandwich	59 (81.9)	2 (3.4)	5 (8.5)	20 (33.9)	30 (50.9)	0	2 (3.4)
Mutton ribs	60 (83.3)	2 (3.3)	3 (5.0)	17 (28.3)	36 (60.0)	1 (1.7)	1 (1.7)
Roasted mutton meat	60 (83.3)	2 (3.3)	6 (10.0)	17 (28.33)	34 (56.7)	0	1 (1.67)
Hind leg	57 (79.2)	1 (1.8)	6 (10.53)	15 (26.3)	33 (57.9)	0	2 (3.5)
Intestines (Achii)	53 (73.6)	1 (1.9)	4 (7.6)	17 (32.1)	28 (52.8)	2 (3.8)	1 (1.9)
Liver	53 (73.6)	1 (1.9)	3 (5.7)	13 (24.5)	30 (56.6)	4 (7.6)	1 (1.9)
Heart	45 (62.5)	1 (2.2)	3 (6.7)	11 (24.4)	25 (55.6)	3 (6.7)	2 (4.4)
Kidneys	47 (65.3)	1 (2.1)	2 (4.3)	11 (23.4)	26 (55.3)	5 (10.6)	2 (4.3)
Lungs	38 (52.8)	1 (2.6)	2 (5.3)	8 (21.1)	22 (57.9)	4 (10.5)	1 (2.6)
Esophagus	18 (25.0)	0	0	4 (22.2)	8 (44.4)	4 (22.2)	2 (11.1)
Hoof	15 (20.8)	1 (6.7)	1 (6.7)	1 (6.7)	8 (53.3)	2 (13.3)	2 (13.3)
Skin	12 (16.7)	0	0	2 (16.7)	5 (41.7)	3 (25.0)	2 (16.7)
Head	46 (63.9)	1 (2.2)	4 (8.7)	11 (23.9)	24 (52.2)	5 (10.9)	1 (2.2)
Tongue	39 (54.17)	1 (2.6)	4 (10.3)	6 (15.4)	22 (56.4)	5 (12.8)	1 (2.6)
Eyes	39 (54.2)	1 (2.6)	4 (10.3)	7 (18.0)	21 (53.9)	4 (10.3)	2 (5.1)
Ears	24 (33.3)	1 (4.2)	3 (12.5)	2 (8.3)	13 (54.2)	4 (16.7)	1 (4.2)
Stomach	45 (62.5)	1 (2.2)	3 (6.7)	12 (26.7)	22 (48.9)	6 (13.3)	1 (2.2)

* Percent is of the number of participants who consumed the respective part of the sheep.

## Supplementary Materials

The following are available online at https://www.mdpi.com/1660-4601/16/21/4195/s1, Lister thesis Chapter 3 and approval documentation.
