# Supplementary material for: Traditional Sheep Consumption by Navajo People in Cameron, Arizona
Source: Int J Environ Res Public Health. 2019 Oct 30;16(21):4195. doi: 10.3390/ijerph16214195 (PMC6862166; doi:10.3390/ijerph16214195)
Supplement: Supplementary file 1 [file ijerph-16-04195-s001.pdf]

CHAPTER 3

THE BIOACCUMULATION OF URANIUM IN SHEEP HEART AND KIDNEY: THE IMPACT OF CONTAMINATED TRADITIONAL FOOD SOURCES ON THE NAVAJO RESERVATION

By Andee R. Lister

A Thesis

Submitted in Partial Fulfillment

of the Requirements for the Degree of Master of Arts

in Sustainable Communities

Northern Arizona University

May 2018

Approved:

Jani C. Ingram, Ph.D., Chair

Margaret M. Briehl Ph.D.

Diana Stuart Ph.D.

Chapter Three

Results and Discussion

3.1 Bioaccumulation of Uranium in Sheep Hearts from Cameron and Leupp

Sheep heart tissue samples were collected from the Cameron and Leupp area to be analyzed by the ICP-MS for uranium content. Five sheep from each chapter, for a total of ten sheep, were euthanized and from each heart a subset of five samples were collected to establish 25 individual samples from Cameron and 25 individual samples from Leupp, for a total of 50 samples. The individual sheep hearts were collected in the field, summer 2013, and then prepped at an analytical chemistry laboratory at NAU. Sample preparation included, slicing tissue samples as thin as possible, hanging them up to dry on cotton clothes line (Household Essentials) for two to three weeks in the prep lab. Each individual heart organ was chopped into smaller dried bits, powdered in a standard coffee grinder (Kitchen Aid), homogenized in a sterile 500 mL Whirl-Pak sample bag (VWR), and then separated into 5 replicates. From each individual homogenized sample, 1.47 g of sample was exacted and placed in sets of five within 10.0 mL ceramic crucibles (VWR). The samples were mineralized in a Thermo Lindberg/Blue M furnace for 24 hours removing the organic matter. Roughly 40 to 60 mg of mineralized tissue sample were transferred to 45 mL centrifuge tube for partial acid digestion, filtration, dilution, and analysis with the ICP-MS. Uranium concentrations within the tissues were calculated in nanograms (ng) per gram (g) of mineralized heart sample.

A total of ten sheep hearts, five sheep hearts from Cameron, and five sheep hearts from Leupp were analyzed. Figure 3.1 demonstrates, the uranium concentrations were below 100 ng/g, but above 50 ng/g. The Cameron heart samples exceeded 65 ng/g except sheep two. Organizing

the heart samples in decreasing concentration of uranium, sheep one (91.5 ng/g), sheep three (87.8 ng/g), sheep four (72.0 ng/g), sheep five (65.3 ng/g), and sheep two (63.3 ng/g), shows that sheep two had the lowest concentration 63.5 ng/g and sheep one had the highest concentration 91.5 ng/g. The range, median, and mean for the Cameron samples were 28.0 ng/g, 72.0 ng/g, and 76.0 ng/g.

The Leupp heart samples exceeded 60 ng/g uranium concentration except sheep five. Ordering the tissues in descending concentration, sheep three (91.5 ng/g), sheep one (76.0 ng/g), sheep four (69.8 ng/g), sheep two (60.5 ng/g) and sheep five (58.7 ng/g), sheep five had the lowest concentration 58.7 ng/g and sheep three had the highest concentration at 91.5 ng/g. The range, median, and mean for the Leupp samples were 32.9 ng/g 75.6 ng/g and 71.2 ng/g.

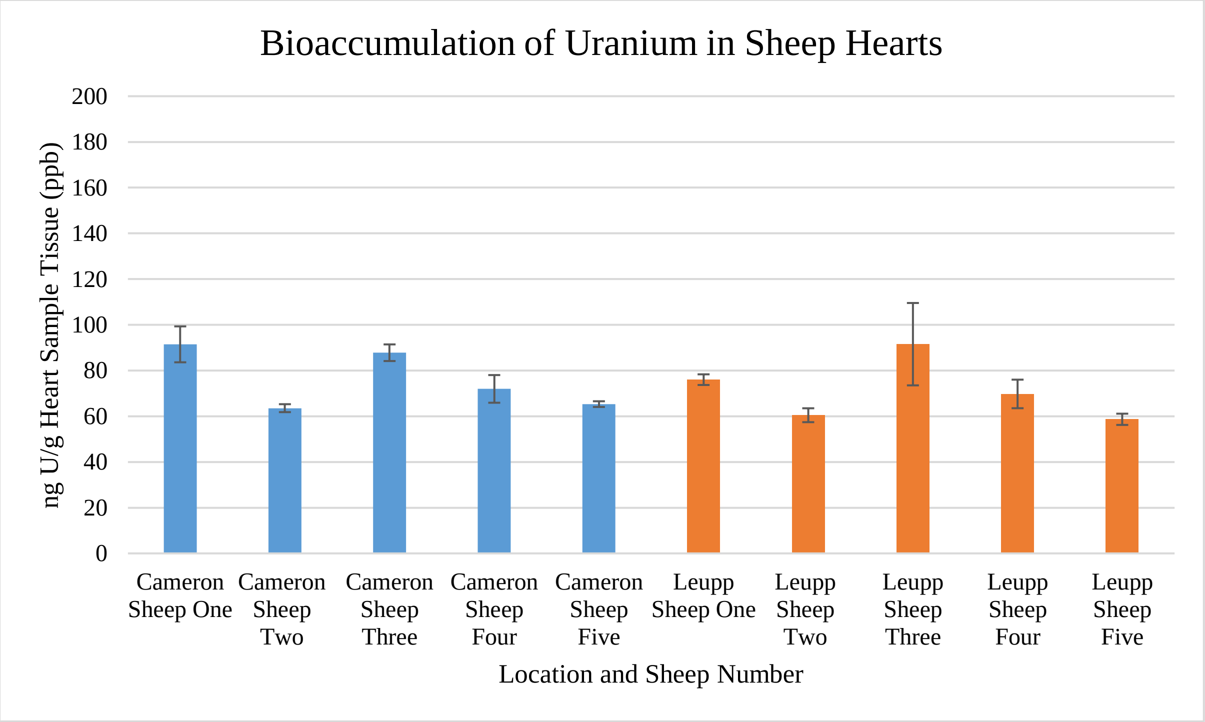

Figure 3.1. Illustrates the uranium concentration in sheep hearts from Cameron and Leupp, AZ. Leupp and Cameron Sheep

Comparing the results and looking at the overall average of each chapter (Figure 3.2), the uranium concentrations from the Cameron and Leupp samples look similar. The overall average of Cameron was 76.0 ng/g and the overall average of Leupp was 71.3 ng/g.

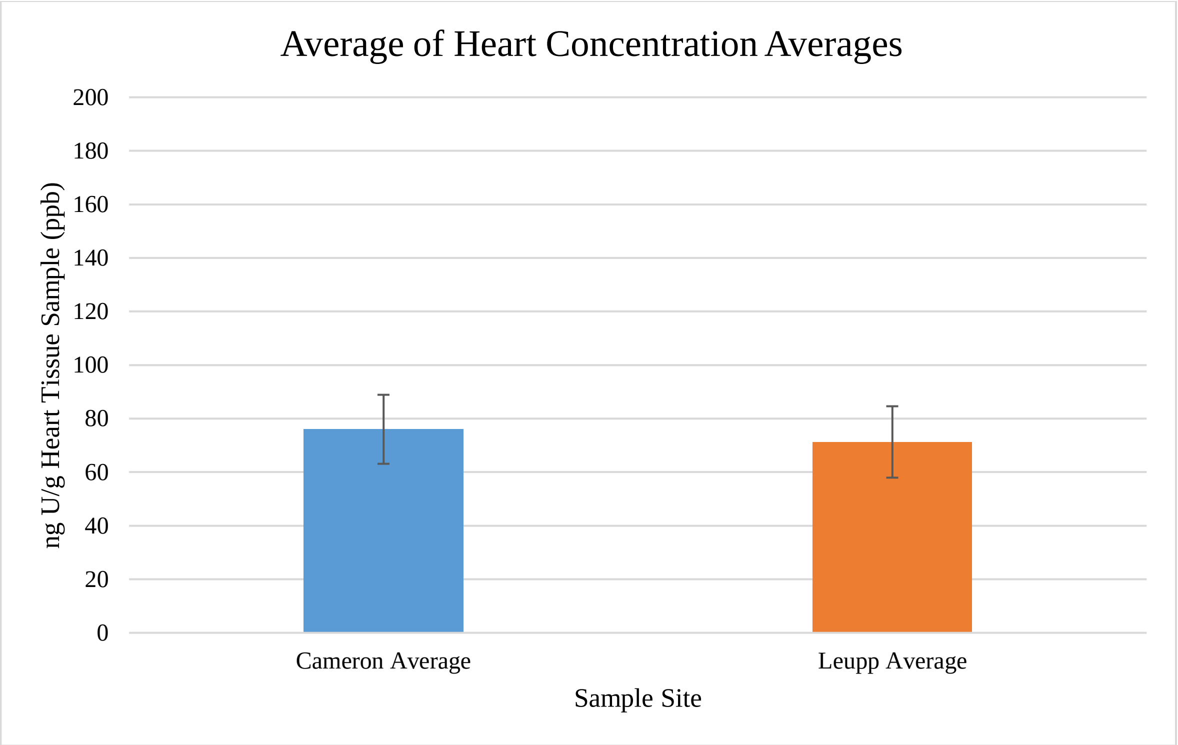

Figure 3.2. Illustrates the average of the averages between Cameron and Leupp, AZ heart samples.

Due to the similar uranium averages from the study areas, a follow up experiment was conducted to determine if the mineralization method was reproducible and/or if this method contributed to the final result. The lack of a standard reference material (SRM), further necessitated this experiment in order to perform a quality assurance/quality control on the preparation protocol. The relative standard deviation (RSD) was calculated for each subset of ten sample to determine if the samples that were compared had an RSD less than ten percent. An RSD less than ten percent would suggest the furnace method was ashing the samples evenly and reproducible. The heart samples were collected, prepared for analysis, and analyzed following the same previous protocol described above. The only change was the fact that the samples were separated into ten replicates, instead of five. This separation was done to decrease the amount of error or variance, in that the more samples the smaller the variance. Figure 3.3 illustrates heart samples from one sheep from Cameron and one sheep from Leupp. This calculation showed

Cameron had an RSD of 5.6 % and the Leupp sheep heart an RSD 7.3% which verified the ashing protocol previously suggested by Lydia Edgewater.

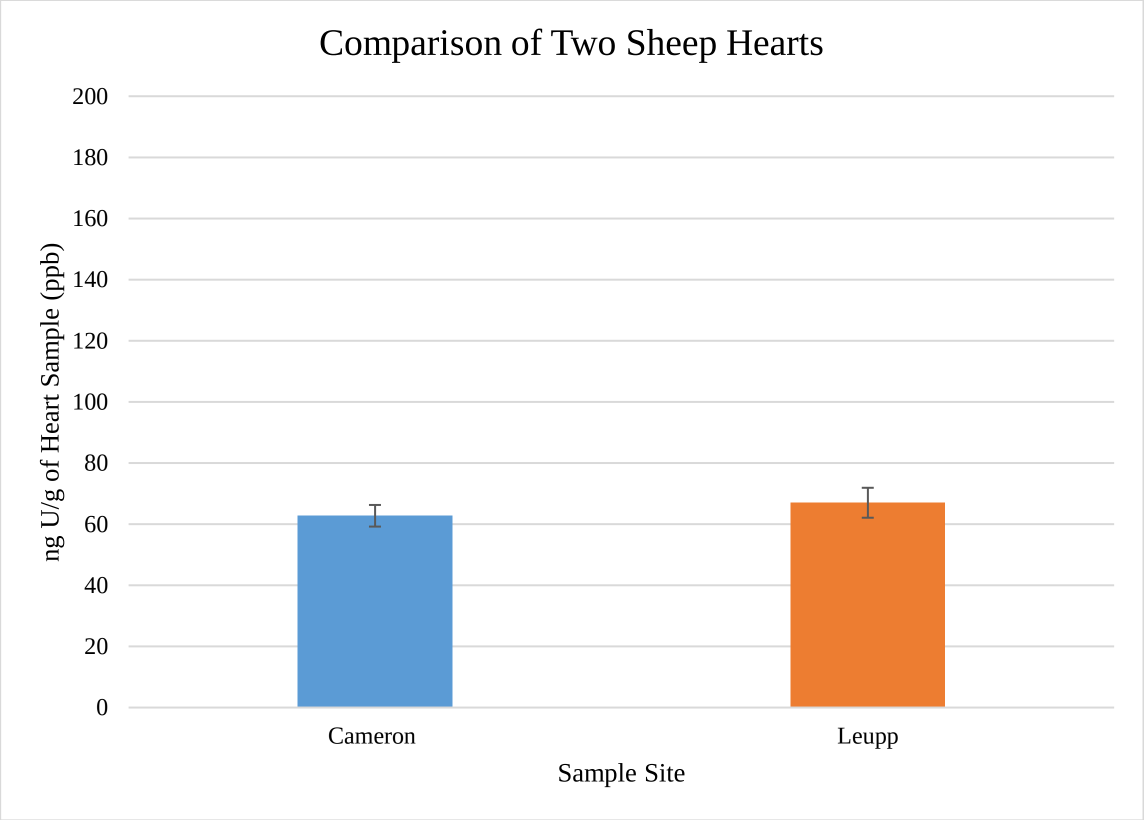

Figure 3.3. Comparison of sample preparation method reproducibility with respect to using the muffle furnace.

3.2 Characterization of Uranium in Sheep Kidneys from Cameron and Leupp

Sheep kidney samples from Cameron and Leupp area were analyzed for uranium concentrations by ICP-MS. Five sheep from each chapter, for a total of ten sheep, were euthanized and from each kidney a subset of three samples were collected to establish 15 individual samples from Cameron and 15 individual samples from Leupp, for a total of 30 samples. Kidney samples were collected upon extraction, prepped at an analytical chemistry laboratory at NAU where they were sliced as thin as possible and allowed to dry for two to three weeks by hanging them up to dry on cotton clothes line (Household Essentials). After drying,

each individual kidney organ was chopped into smaller dried bits, powdered in a standard coffee grinder, and homogenized in a sterile gallon sized sample bag (Hefty). Approximately one to two grams of homogenized sample were filled in sets of three into ten mL vials. The samples were mineralized in a Thermo Lindberg/Blue M furnace for 24 hours removing the organic matter. Roughly, 90 to 40 mg of mineralized sample was transferred to 45 mL centrifuge tubes for acid digestion, filtration, and dilution, and analysis by ICP-MS.

Figure 3.4 illustrates average uranium concentration values measured in sheep kidneys collected from Cameron and Leupp during summer 2013. Uranium content in the tissue was calculated in nanograms (ng) of uranium per gram (g) of mineralized kidney sample. The Cameron kidney samples exceeded 98.0 ng/g with an exception of sheep three. Organizing the kidney samples in decreasing concentration of uranium, sheep one (199.3 ng/g ng/g), sheep two (198.9 ng/g), sheep five (100.8 ng/g), sheep four (98.0 ng/g), and sheep three (5.5 ng/g), sheep three had the lowest concentration at 5.5 ng/g, while sheep one had the highest concentration at 199.3 ng/g. The range, median, and mean for the Cameron samples was 193.8 ng/g, 100.8 ng/g, and 120.5 ng/g. The Leupp kidney samples exceeded 80 ng/g with an exception of sheep three. Organizing the kidney samples in decreasing concentration of uranium, sheep one (188.3 ng/g), sheep two (159.2 ng/g), sheep five (124.2 ng/g), sheep four (89.8 ng/g), and sheep three (70.9 ng/g), sheep three had the lowest concentration at 70.9 ng/g, while sheep one had the highest concentration at 188.3 ng/g. The range, median, and mean for the Leupp samples was 117.4 ng/g, 124.2 ng/g, and 126.5 ng/g.

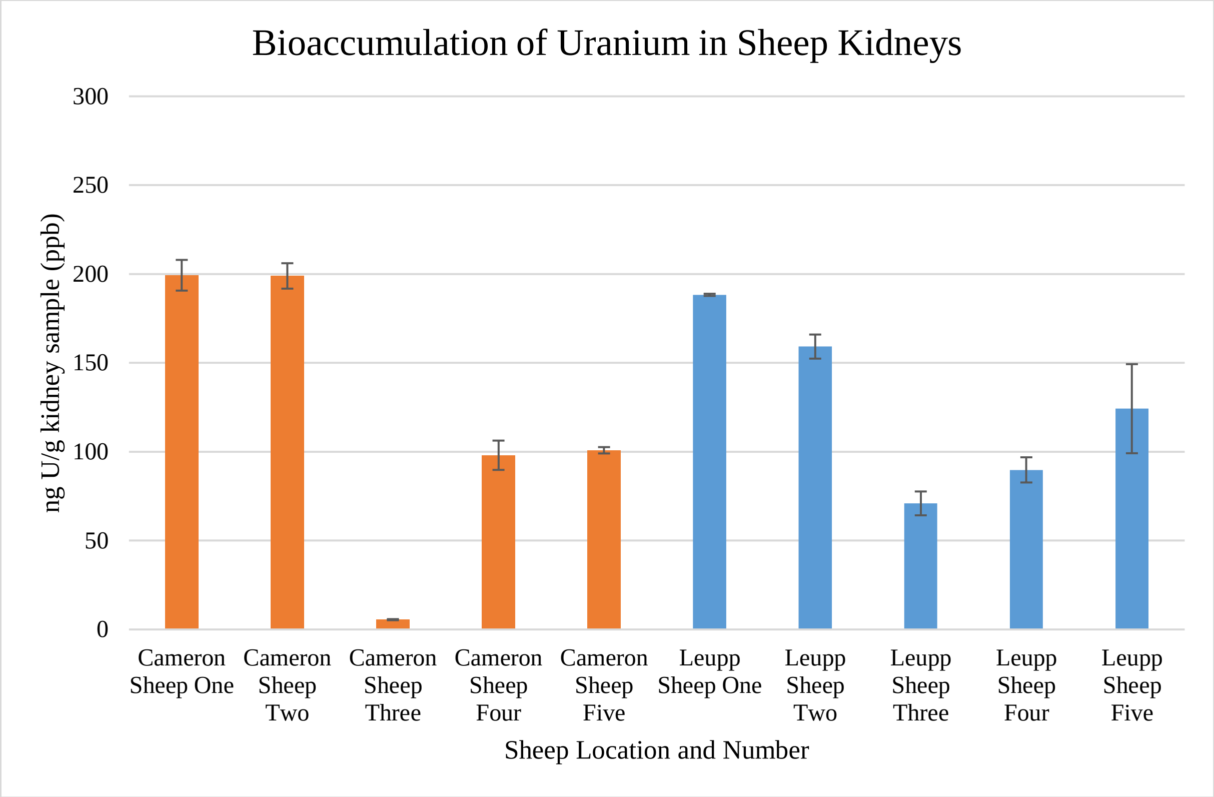

Figure 3.4. Uranium concentrations in sheep kidneys from Cameron (mining site of interest) and Leupp (control site) in ppb.

Comparing the results and looking at the overall average of each chapter (Figure 3.5), Cameron and Leupp look relatively similar. The overall average of Cameron samples was 120.5 ng/g and the overall average of Leupp samples was 126.5 ng/g. As a result, of the similarities in averages observed in the Cameron and Leupp heart and kidney samples, as well as verifying the sample preparation method, an additional control site was added to the thesis project to answer the question of "Do the Cameron and Leupp heart and kidney samples have uranium levels that are at background or elevated?".

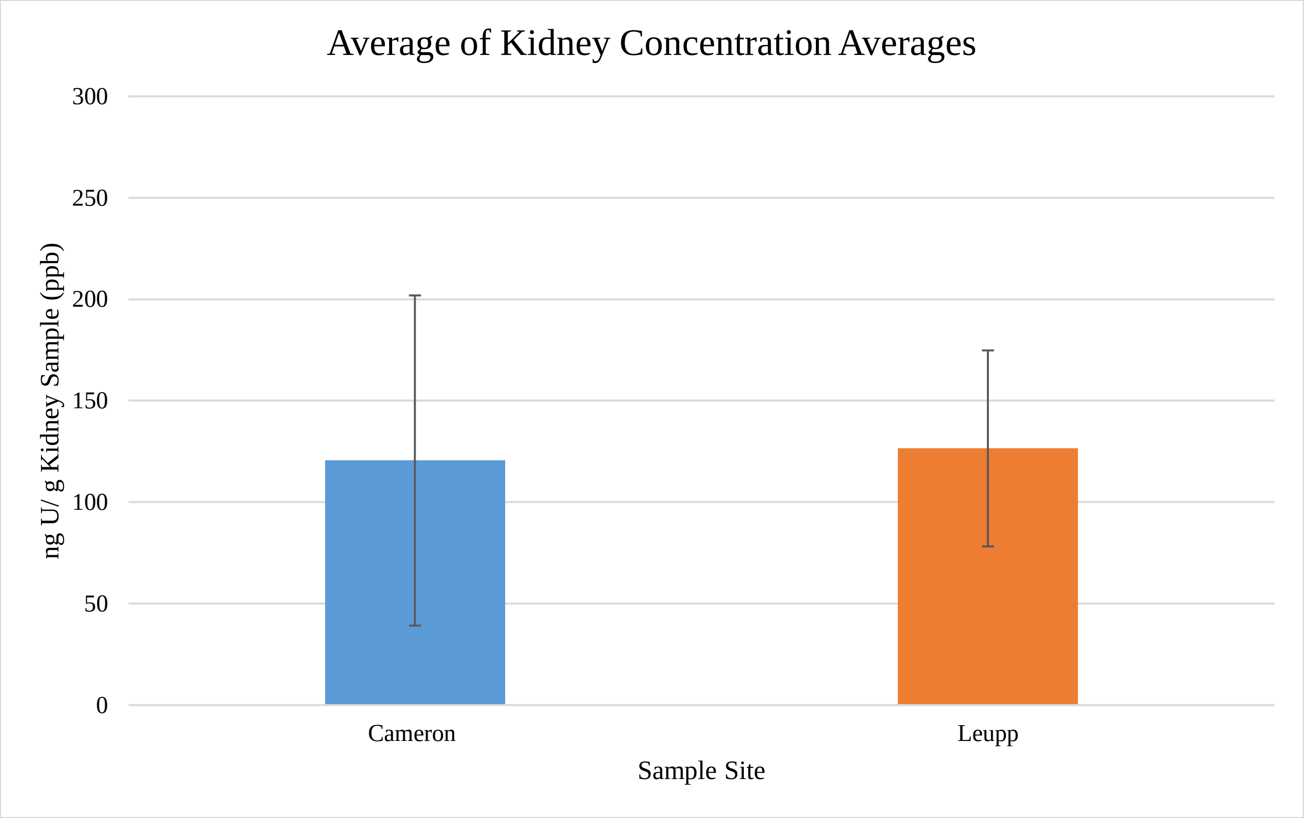

Figure 3.5. Average of the average kidney concentration from both Cameron and Leupp.

3.3 Characterization of Uranium in Sheep Kidneys from Eagar, Cameron, and Leupp, AZ

Sheep kidney tissue samples were collected from the Cameron, Leupp, and Eagar area to be analyzed by the ICP-MS for uranium content. Five sheep from each chapter or community, for a total of 15 sheep, were euthanized and from each kidney a subset of three samples were collected to establish 15 individual samples from Cameron, 15 individual samples from Leupp, and 15 individual samples from Eagar, for a total of 45 samples. The sheep from Eagar were collected and euthanized roughly five miles south of downtown Springerville, AZ, and roughly 200 miles southeast of the Navajo reservation (Figure. 3.6). The collaboration was done with the assistance of a private farmer Jessie Thomas, whom we were put into contact through a former student of Dr. Ingram's. The Eagar sheep were kept in a corral, and there will be a follow up to

get information on what type of diet the sheep had and what type of water the sheep drank from regularly.

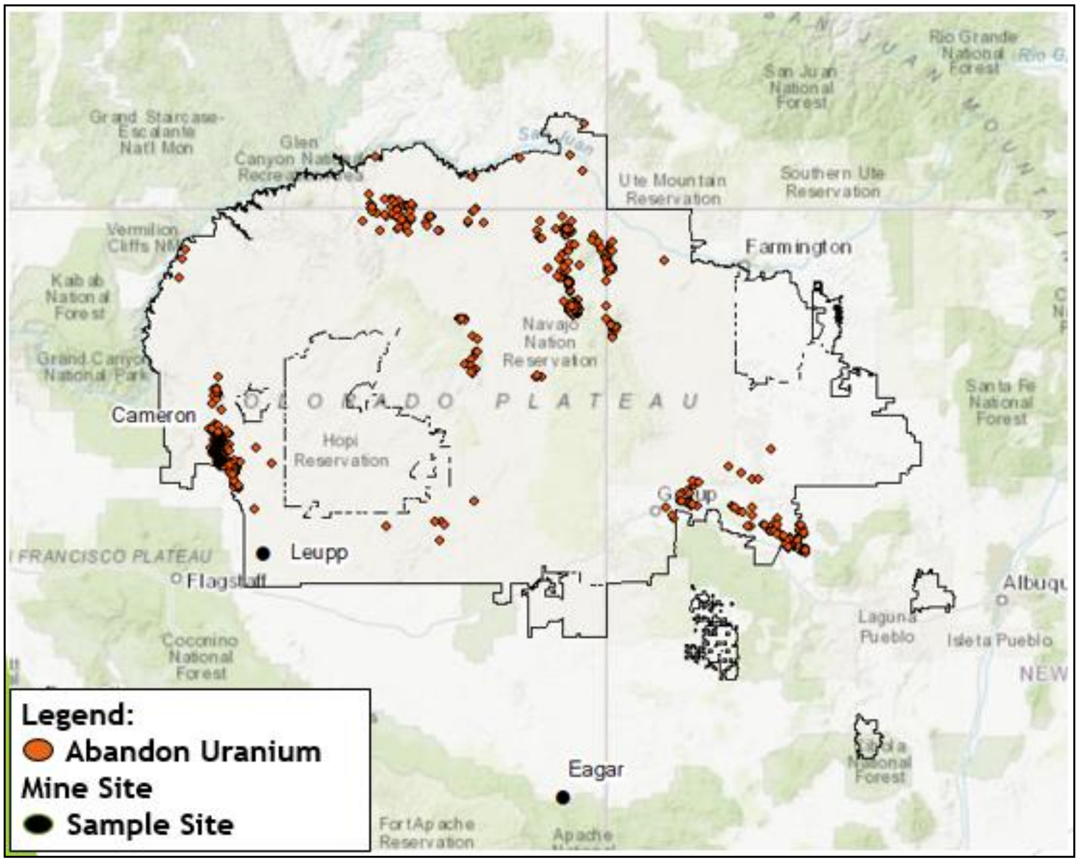

Figure 3.6. Map of all sites sampled

The Eagar kidney samples were collected, prepared for analysis, and analyzed following the same previous protocol, except, roughly 200 to 300 mg of mineralized sample was transferred to 45 mL instead of 90 to 40 mg of sample. Figure 3.7, demonstrates the uranium concentrations in kidneys from Cameron, Leupp, and Eagar were below 200 ng/g and above 5 ng/g.

The Eagar kidney samples exceeded 20 ng/g. In decreasing order, sheep two (45.5 ng/g ng/g), sheep one (37.8 ng/g), sheep five (28.8 ng/g), sheep three (25 ng/g), and sheep four (23.4 ng/g). Sheep four had the lowest concentration at 23.4 ng/g, while sheep two had the highest

concentration at 45.5 ng/g. The range, median, and mean for the Eagar kidney samples were 22.1 ng/g, 28.8 ng/g, and 32.1 ng/g.

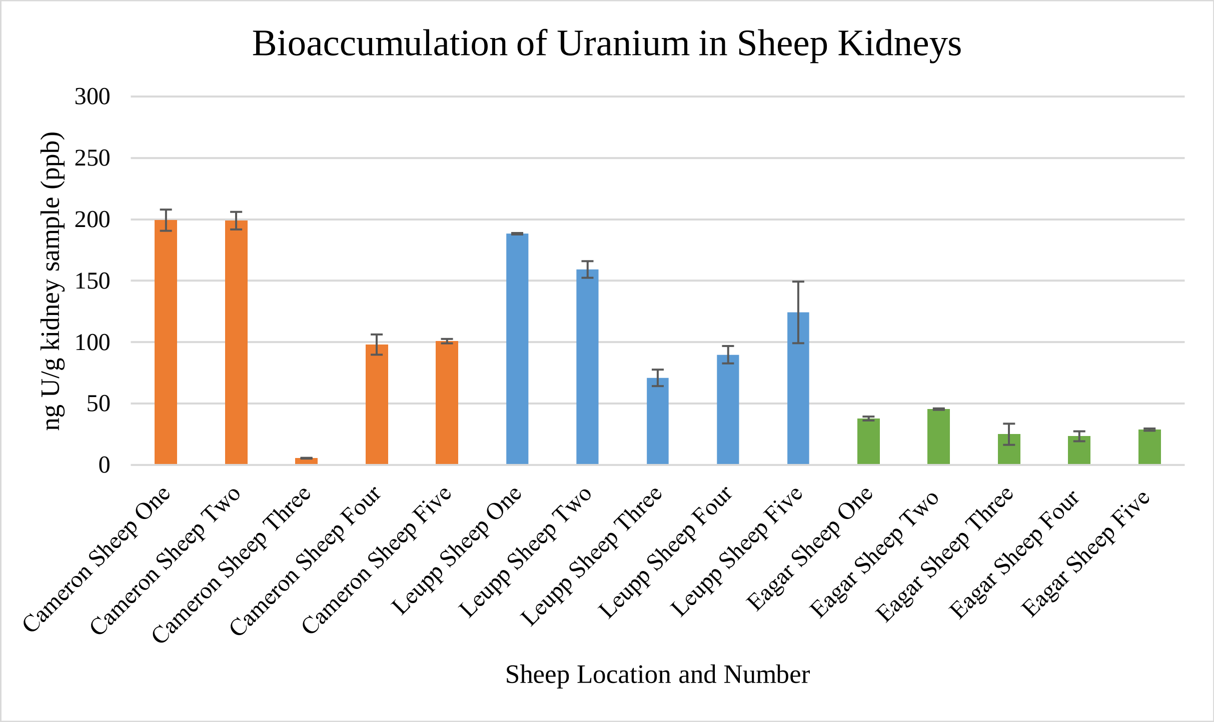

Figure 3.7. Illustrates the uranium concentrations from Cameron, Leupp, and Eagar

The Cameron kidney samples exceeded 98 ng/g of uranium with an exception of sheep three, sheep three had the lowest uranium concentration at 5.5 ng/g, sheep one had the highest uranium concentration at 199.3 ng/g. The Leupp kidney samples exceeded 80 ng/g with an exception of sheep three, sheep three had the lowest uranium concentration at 70.9 ng/g, sheep one had the highest uranium concentration at 188.3 ng/g.

Focusing on the overall average of each chapter or community, Cameron and Leupp look relatively similar, and Eagar results are much lower than the results from both Cameron and Leupp. The average of the averages was graphed and confirmed both Cameron and Leupp results are relatively similar (Fig. 3.6), while Eagar is lower than Cameron and Leupp. The overall

average of Cameron samples was 120.5 ng/g, the overall average of Leupp samples was 126.5 ng/g, and the overall average of the Eagar samples was 32.1 ng/g

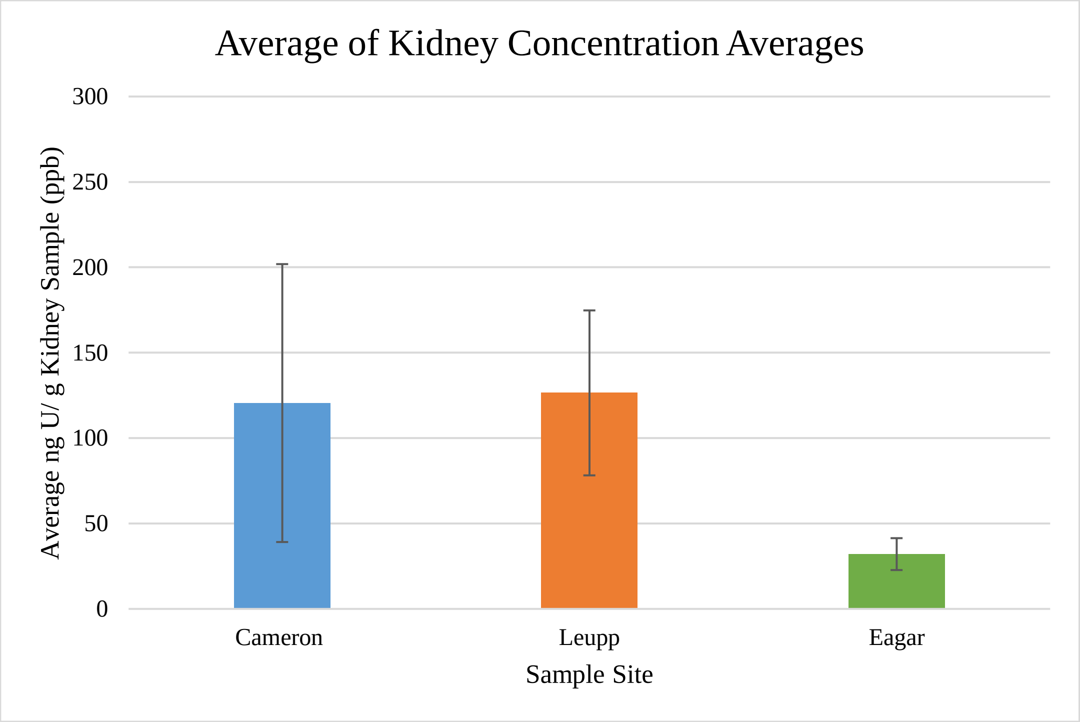

Figure 3.8. Uranium concentrations in sheep kidneys from Cameron (mining site of interest), Leupp (initial control site), and Eagar, AZ (overall control) in ppb.

3.4 Discussion

The methods for this project were being developed with the Cameron and Leupp samples, which is the reason why there is such a large range in the amount of mineralized sample return. Through the previous method development with the Cameron and Leupp samples, it was established that nine grams of powdered tissue sample mineralizes down to 300 mg of ashed sample. This established protocol was followed during the Eagar sample preparation and showed the larger amount of sample improved analysis by improving efficiency, reproducibility, and reducing method error. Establishing this protocol was important to decrease the amount of variation between samples from one organ and increase throughput for analysis.

In the thesis, only the heart and kidney results are reported, although other tissues were collected for future studies and analysis. Sheep kidney samples from Cameron, AZ was analyzed and had an average concentration of 120.5 ng/g, the lowest and the highest concentration were observed at 5.5 and 199.3 ng/g respectively for sheep three and sheep one. Sheep three had the lowest concentration among the Cameron and Leupp samples, which suggests gross error during sample preparation. Additional analysis could not be conducted to validate the Cameron sheep samples, due to limited sample and method development, therefore a Grubbs test will be conducted to determine if sheep three from Cameron is an outlier. Sheep kidney samples from Leupp, AZ were analyzed and had an average concentration of 126.5 ng/g. The lowest and the highest concentration were observed at 70.9 and 188.3 ng/g respectively for sheep three and sheep one. Both Cameron and Leupp sheep kidney results showed similar concentrations although they were expected to be different, since the Cameron flock came from an area with a history of uranium mining and the Leupp flock had no history of uranium mining. Based on the inconclusiveness of Cameron and Leupp sheep kidneys, an additional control site was added from Eagar, AZ. Eagar is not on the Navajo Nation, but roughly 200 miles southeast of the Navajo Nation. Sheep kidney samples from Eagar, AZ were analyzed and had an average concentration of 32.1 ng/g. The lowest and highest concentration was observed at 23.4 and 45.5 ng/g respectively for sheep four and sheep two. The Eagar sheep illustrated lower concentrations than Cameron and Leupp, suggesting that the reservation sheep (Cameron and Leupp) have a much higher background concentration than the off the reservation sheep (Eagar).

A two-sided t-test and 95% confidence interval confirmed that Cameron and Leupp were not equal to each other, and there is not a significant difference between the two concentrations. The inconclusiveness is most likely due to the high standard deviation from both Cameron and Leupp. Additional statistics indicated that the Navajo reservation sites (Cameron and Leupp) are not equal to Eagar, and the reservation sites were statistically higher than Eager. Possible reasons for this statistical significance is weather patterns and the dispersion of dust particulates over a certain area, in that Cameron and Leupp are roughly 40 miles apart from each other. The other possible reason is that people often trade sheep from Cameron and Leupp, and vice versus. Again, these are just two possibilities, though there are other variables that should be considered and evaluated.

A t-test was used to compare one mean value to another to decide whether there was a statistically significant difference between two means. Focusing the difference of means between sheep hearts and kidneys from one chapter or community, the t-test confirmed that the sheep from Leupp were significantly different from each other; while Cameron sheep that were taken from a flock were similar (i.e. sheep one and three were similar, sheep two and five were similar, and sheep four was different from all of the Cameron sheep). The t-test confirmed that the Eagar sheep were significantly different from each other. The reason this t-test was conducted was to see if the sheep coming from one herd or area were similar as they were exposed to the same living conditions. The statistical analysis confirms that biological samples, especially when dealing with grazed animals, require that additional information and measurements, such as lifestyle and location of origin, be gathered to account for error within and between samples.

The heart is a muscle that pumps blood throughout the body and represents a site where uranium is not expected to accumulate. This is a possibly reason why you see there are similar concentrations between Leupp and Cameron. The kidney is required and responsible for maintaining total body salt, water, potassium, and acid-base balance, while eliminating waste products and toxins, and preserving the body's internal environment.[46] The kidney is also recognized as a major site for uranium accumulation, which is why it was expected to see elevated concentrations of uranium in the kidney compared to the heart. This is also a possible explanation for the observation that sheep on the reservation had a higher concentration of uranium than sheep off of the reservation.

One of the many limitations of the study was the unknown ages of the sheep, instead an estimation was made by the veterinarian on site. The veterinarian mentioned that if the sheep was grazing in an arid desert like area like Cameron that the teeth would wear much more and not be a great indicator of age. The reason why this was important is because the older the sheep, the longer they have the potential to be exposed to uranium and suffer any damage to uranium. The second limitation, the number of sheep sampled is not statistically significant and it can only be used as a statistical simulation, in that we have a sample size of five from each site. Statistical consultants have suggested collecting more sheep (n= 30 from each site); however, that would have proven difficult as the community members in Cameron have decreased the amount of sheep they raise as they do not know how their sheep are being affected by the legacy of uranium mining. The third limitation, the history of each sheep was unknown, in that it was unknow if the sheep was grazing in certain areas for most of their lives, or if they were traded and moved from one community to the next. Understanding the area from where the sheep originated is important because the Navajo Nation represents a large land mass with varying environmental conditions

and differing environmental contaminants, uranium mining is variable. Furthermore, if the sheep come from a site off of the reservation, this represents another set of possible exposures from where they came from. Additionally, the areas in which the Leupp sheep grazed was unknown, the rancher disclosed the details that he did have a summer and winter grazing area. The fourth limitation, an SRM for uranium in biological tissues was absent, and the search for an SRM for this specific project is still ongoing. The reason an SRM was important is to establish and ensure the accuracy and reliability of the measurements of the tissues that were analyzed.

THE NAVAJO NATION

RUSSELL BEGAYE PRESIDENT
JONATHAN NEZ VICE PRESIDENT

February 21, 2017

Jani C. Ingram, Ph.D.
Northern Arizona University
P.O. Box 5698
Flagstaff, Arizona 86011

Dear Dr. Ingram,

This is to advise you that the **Study #NNR-17.266T: "Mutton Consumption Survey"** has been presented to the Navajo Nation Human Research Review Board (NNHRRB) on **February 21, 2017**, and the following action taken subject to the conditions and explanation provided below.

Reasons:	New Application
Description:	Request Review and Acceptance of New Protocol
**NNHRRB Action:**	**Accepted and Approved**
Conditions:	From February 21, 2017 – February 21, 2018 period With All Standard Conditions

The Navajo Nation Human Research Review Board has added a very important additional contingency regarding failure to comply with NNHRRB rules, regulations, and submittal of reports which could result in sanctions being placed against your project. This could also affect your funding source and the principal investigator. Under Part Five: Certification, please note paragraph five wherein it states: *"I agree not to proceed in the research until the problems have been resolved or the Navajo Nation Human Research Review Board has reviewed and approved the changes."* Therefore, it is very important to submit quarterly and annual reports on time and if continuation is warranted submit a letter of request sixty (60) days prior to the expiration date.

The following are requirements that apply to all research studies:

1. The Navajo Nation retains ownership of all data obtained within its territorial boundaries. The Principal Investigator shall submit to the NNHRRB a plan and timeline on how and when the data/statistics will be turned over to the Navajo Nation;
2. Only the approved informed consent document(s) will be used in the study;
3. Any proposed future changes to the protocol or the consent form(s) must again be submitted to the Board for review and approval prior to implementation of the proposed change;
4. If the results of the study will be published or used for oral presentations at professional conferences, the proposed publication, abstract and/or presentation materials must be submitted to the Navajo Research Program for Board review and prior approval;
5. Upon Board approval, three (3) copies of the final publication must be submitted to the Navajo Research Program;
6. All manuscripts must be submitted to the Navajo Research Program for Board Review and prior approval;
7. The Principal Investigator must submit a dissemination plan on how the results of the study and how these results will be reported back to the Navajo Nation;

8. The Principal Investigator must share specifically how these results will generally benefit or improve the health of the Navajo people. This can be completed by:
   a. Conducting an educational in-service for the community people and health care providers on the Navajo Nation and present the findings. Provide documentation of these in-services presented.
   b. Developing educational materials for use by the health care providers and the community people and providing the training on how to use the materials; and
   c. Presenting and sharing the results of the study at a research conference sponsored by the Navajo Nation for its health care providers and the Navajo people.
9. The Principal Investigator is expected to submit documentation on 8a, b, & c;
10. The Principal Investigator must submit quarterly and annual reports as scheduled.

Please begin using your Study Protocol Number **NNR-17.266** on all correspondences. If you have any questions on this subject, please call the Navajo Research Program at (928) 871-6929.

Sincerely Yours,

Beverly Becenti-Pigman, Chairperson
Navajo Nation Human Research Review Board

cc: #NNR-17.266 file

NORTHERN ARIZONA UNIVERSITY

Institutional Review Board for the
Human Research Subjects Protection

128 South Knolls Dr
Building 56, suite 240
PO Box: 4087
Flagstaff AZ 86011
928-523-9551
928-523-1607 fax
http://nau.edu/Research/Compliance/Human-Subjects/Welcome

**To:**	Jani Ingram, PhD
**From:**	NAU IRB Office
**Approval Date:**	February 2, 2017
**Project:**	Mutton Consumption Survey
**Project Number:**	1009330-1
**Submission:**	New Project
**Review Level:**	Exempt Review
**Action:**	APPROVED WITH CONDITIONS
**Project Status:**	Exempt
**Review Category/ies:**	**Exempt Approval 45 CFR 46.101(b)(2):** Research involving the use of educational tests (cognitive, diagnostic, aptitude, achievement), survey procedures, interview procedures or observation of public behavior.<br><br>**Condition to Approval:** Once it is obtained, submit a copy of the Navajo Nation Human Research Review Board approval for NAU IRB acknowledgement prior to engagement in research.

This submission meets the criteria for exemption under 45 CFR 46.101(b). This project has been reviewed and approved by an IRB Chair or designee.

- Northern Arizona University maintains a Federalwide Assurance with the Office for Human Research Protections (FWA #00000357).
- All research procedures should be conducted in full accordance with all applicable sections of the guidance.
- Exempt projects do not have a continuing review requirement.
- This project should be conducted in full accordance with all applicable sections of the guidance and you should notify the IRB immediately of any proposed changes that affect the protocol.
- Amendments to exempt projects that change the nature of the project should be submitted to the Human Research Subjects Protection (HRSP) office for a new determination. See the guidance Exempt Research for more information on changes that affect the determination of exemption. Please contact the HRSP to consult on whether the proposed changes need further review.
- You should report any unanticipated problems involving risks to the participants or others to the IRB.
- All documents referenced in this submission have been reviewed and approved. Documents are filed with the HRSP Office. If subjects will be consented, the approved consent(s) are attached to the approval notification from the HRSP Office.

Generated on IRBNet
